# Prevalence of and Factors Associated with Overactive Bladder, Anxiety, and Depression Among Patients with Multiple Sclerosis: A Cross-Sectional Study in Saudi Arabia

**DOI:** 10.3390/clinpract16060114

**Published:** 2026-06-15

**Authors:** Mohammed Alqwaifly, Samer A. Almuqairsha, Emad Alwashmi, Yousef M. Alharbi, Adi A. Aldubaiyan, Raghad H. Aldligan, Abdulmajeed A. Alkhamees, Ayham Abazid, Rehana Khalil, Osama Al-Wutayd

**Affiliations:** 1 Department of Medicine, College of Medicine, Qassim University, Buraydah 52571, Saudi Arabia; s.almuqersha@qu.edu.sa; 2 Department of Surgery, College of Medicine, Qassim University, Buraydah 52571, Saudi Arabia; e.alwashmi@qu.edu.sa; 3 Department of Neurology, King Fahad Hospital of the University, Al Khobar 31952, Saudi Arabia; ymalharbi@iau.edu.sa; 4 Neuroscience Department, King Faisal Specialist Hospital & Research Centre, Riyadh 11211, Saudi Arabia; f1524925@kfshrc.edu.sa; 5 Department of Neurology, King Saud Medical City, Riyadh First Health Cluster, Riyadh 12991, Saudi Arabia; raghadaldligan@rfhc.gov.sa; 6 Department of Psychiatry, College of Medicine, Qassim University, Buraydah 52571, Saudi Arabia; a.alkhamees@qu.edu.sa; 7 Department of Medicine, King Fahad Specialist Hospital, Qassim, Buraydah 51451, Saudi Arabia; aabazed@moh.gov.sa; 8 Department of Family and Community Medicine, College of Medicine, Qassim University, Buraydah 52571, Saudi Arabia; rn.noman@qu.edu.sa

**Keywords:** prevalence, depression, anxiety, overactive bladder, multiple sclerosis, Saudi Arabia

## Abstract

Background: Over the years, it has become increasingly clear that neurological conditions, such as multiple sclerosis (MS), commonly exhibit other health problems. Therefore, this is the first study aimed at investigating the prevalence of and factors associated with three binary outcomes: depression, anxiety, and an overactive bladder (OAB) among MS patients in the Qassim region, Saudi Arabia. Methods: This cross-sectional study was conducted in the neurological department of King Fahad Specialist Hospital in the Qassim region, Saudi Arabia, from January to December 2024. Data on age, sex, marital status, occupation, body mass index (BMI), MS duration, comorbidities, anxiety, depression, and OAB symptoms (frequency, nocturia, urgency, and urge incontinence) were obtained. Results: Of the 262 MS patients in this study, 184 (70.2%) were females, and 78 (29.8%) were males. The median values [IQR] of age and MS duration were 34 [26–40] and 5 [2–9] years, respectively. The prevalence of depression, anxiety, and OAB were 53.4%, 43.9%, and 50%, respectively. Nocturia was the most frequent urinary symptom, and urge incontinence was significantly higher among females. Multiple logistic regression analyses were conducted to assess factors associated with three binary outcomes: depression, anxiety, and OAB. For depression, being single and anxiety were associated with increased risk. Regarding anxiety, being a student was related to decreased risk, while being female and having depression were associated with increased risk. For OAB, only anxiety was associated with increased risk. Conclusions: Approximately one in two MS patients experience either depression or OAB, while anxiety was reported by fewer than half of the patients. This high prevalence of the three outcomes has critical implications for healthcare policy and resource allocation. Thus, screening, early diagnosis, and intervention, as well as integrated care, should be prioritized by healthcare institutions and practitioners to address these conditions and improve MS patients’ quality of life.

## 1. Introduction

Multiple sclerosis (MS) is an autoimmune neurological disorder in which the immune system attacks the brain and spinal cord, impairing cognitive, emotional, sensory, and motor functions [1]. The causes of MS are unknown; however, genetic and infectious diseases and environmental factors, such as vitamin D deficiency, obesity, and smoking, are identified as increasing its risk [2,3]. The prevalence of MS varies globally and is highest in Europe and North America, and there are around 2.8 million MS patients worldwide [4]. The Arabian Gulf countries were marked as belonging to a low-risk zone, but recent data showed a significant increase, ranging from 31 to 55 MS cases per 100,000 individuals in the population [5]. The prevalence of MS in Saudi Arabia is alarming, with regional variation accounting for 61.95/100,000 among Saudi nationals and 40.40/100,000 in the whole population [6]. As a potentially progressive disorder, MS can lead to lifelong disabilities, with impacts on quality of life (QoL) and productivity [7]. A study in the Qassim region, Saudi Arabia, recorded that over 50% of individuals with MS faced difficulties in performing their daily activities, adversely affecting their lifestyles [8]. A wide range of studies reported a high prevalence of urinary tract symptoms among MS patients, including an overactive bladder (OAB), which were more severe in patients with progressive MS and associated with a longer duration and increasing physical disability [9,10,11,12]. A study in Riyadh, Saudi Arabia, showed a high prevalence of lower urinary tract symptoms among MS patients, with significant negative impacts on their QoL [13]. Anxiety and depression are also recognized as prevalent conditions that impair the QoL of patients diagnosed with MS [12,14,15]. Factors such as female gender, younger age, unemployment, short duration since last MS relapse, physical inactivity, past psychiatric history, non-compliance with MS medication, and fatigue have been identified as risk factors related to anxiety [15,16,17]. Notable predictors of depression include male gender, low education, unemployment, physical inactivity, substance use, and fatigue [15,16]. A lower level of social support was a consistent predictor of both anxiety and depression [15]. Some studies in Saudi Arabia also demonstrated that depression and anxiety were significantly associated with MS cases [18,19,20]. Despite a number of studies conducted in some regions in Saudi Arabia, there is a notable gap in the literature regarding the regional variation in the prevalence of MS. The central part of the country, including the Qassim region, has one of the highest rates of MS cases, which should be of particular concern [6]. In addition, potential differences may be attributed to geographic, clinical, and healthcare access factors. The Qassim region is characterized by prolonged dry summer temperatures compared with other regions that have different climate conditions [21], which may influence the severity of MS symptoms. Moreover, advanced tertiary medical cities and MS-specialized physicians are predominantly centralized in Riyadh, Makkah, and the Eastern regions [22,23], which may affect disease presentation, diagnosis, and severity. Furthermore, there is scarce research on OAB symptoms among MS patients in Saudi Arabia, and no data regarding OAB symptoms, depression, and anxiety among MS patients in the Qassim region. Thus, in this study, we aimed to assess the prevalence of and factors associated with OAB, depression, and anxiety among MS patients in the Qassim region.

## 2. Methods

### 2.1. Study Setting and Design

A cross-sectional study was conducted in the neurological department of King Fahad Specialist Hospital, Buraydah, the capital city of Qassim region, located in the center of Saudi Arabia. The only tertiary hospital in the region, it has a >400-bed capacity [24]. Of the region’s population of nearly 1.4 million, on average, around 35% comprises young people between 15 and 34 years old [25].

### 2.2. Data Collection Tool and Method

**Figure 1:**
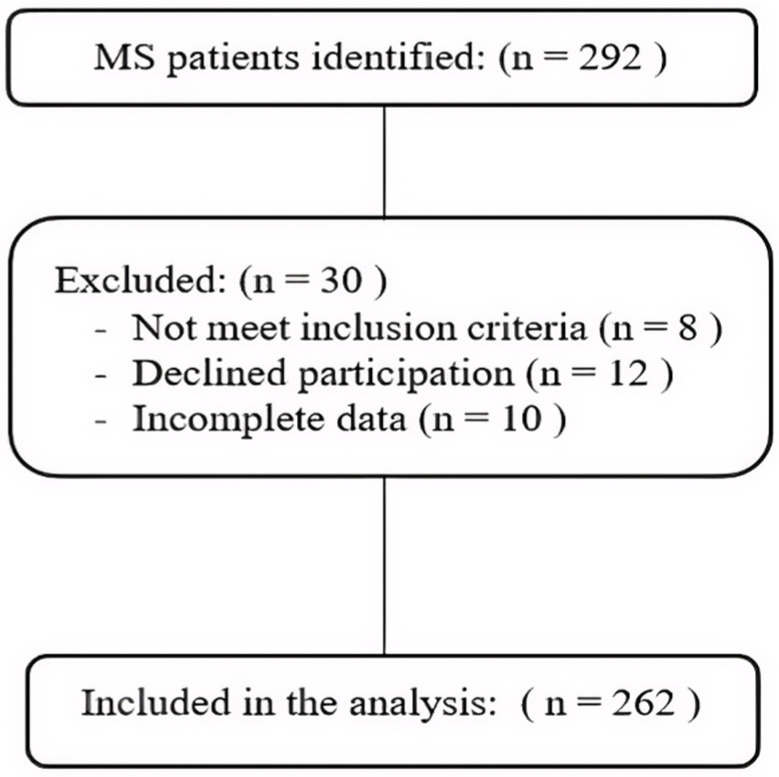
Flowchart of MS patient selection.

Data were retrieved from the medical records of all MS patients who were being treated in neurological clinics from January to December 2024. Data included current age, MS duration, sex, marital status, occupation, body mass index (BMI), and comorbidity. The cases for inclusion in the study had to meet the following criteria: individuals aged 18 years or older with an MS diagnosis confirmed by a neurologist. Those under the age of 18 and who declined to participate were excluded. A self-administered questionnaire was distributed to the participants to assess their OAB symptoms (frequency, nocturia, urgency, and urge incontinence) in accordance with the guidelines of the International Continence Society (ICS), in addition to the Arabic version of the four-item Overactive Bladder Symptom Score (OABSS) questionnaire [26,27], the patient health questionnaire-9 (PHQ-9) for depression, and the General Anxiety Disorder-7 (GAD-7) scale for anxiety [8,28]. Figure 1 outlines the selection process.

### 2.3. Definition of Three Binary Outcomes

The OABSS measures four symptoms (frequency, nocturia, urgency, and urge incontinence) reported over one week. The sum of the scores ranges from 0 to 15, with higher scores indicating greater OAB symptom severity [27]. OAB is defined as ≥3 for the total OABSS and ≥2 for the urgency score [29]. The OAB group is further classified into mild (≤5), moderate (6–11), and severe (≥12) categories [29]. Depression was recorded using the PHQ-9 questionnaire, which is internationally validated [8,28]. Usually, PHQ-9 scores above 5, 10, 15, and 20 are classified as mild, moderate, moderately severe, and severe depression, respectively [30]. For this study, we decided that PHQ-9 scores ≥ 10 should be labeled as positive for depression [31,32]. Anxiety was assessed using the internationally validated GAD-7 scale [8,29]. GAD-7 scores above 5, 10, and 15 are classified as mild, moderate, and severe anxiety, respectively [33]. For the purpose of this study, GAD-7 scores ≥ 10 were classified as positive for anxiety [31,32].

### 2.4. Statistical Analysis

Data were analyzed using STATA software version 16. The number and percentage were calculated for each categorical variable, and the median and the interquartile range (IQR) for each non-normally distributed quantitative variable. Simple logistic regression was conducted, with OAB, anxiety, or depression used as the dependent variable and the associated factors as the independent variables. A multiple logistic regression model was applied to determine the factors associated with OAB, anxiety, or depression. Variables with a *p*-value < 0.25 in the simple logistic regression were included in the multiple logistic regression. The appropriateness of the models and multicollinearity were assessed using the area under the receiver operating characteristic (ROC) curve and the variance inflation factor (VIF), respectively. A *p*-value < 0.05 was considered to be strong evidence against the null hypothesis.

### 2.5. Ethical Considerations

Ethical approval was obtained from the regional Ethics Committee in the Qassim region (Reference no. 607453468) in September, 2023. The methods employed in this study were in accordance with the ethical standards of institutional and national research committees and with the 1964 Helsinki Declaration and its subsequent amendments.

## 3. Results

Of the 262 MS patients enrolled in this study, 184 (70.2%) and 78 (29.8%) were females and males, respectively. The median [IQR] age, MS duration, and OABSS were 34 [26–40] years, 5 (2–9) years, and 4 years (1–7), respectively. Of the 262 MS patients, 137 (52.3%) were married, 146 (55.7%) were unemployed, 84 (32%) and 46 (17.6%) were overweight and obese, respectively, and only 31 (11.8%) had a comorbidity. The prevalence of mild, moderate, and severe anxiety was 31.3%, 21.4%, and 22.5%, respectively. Similarly, the prevalence of mild, moderate, moderate to severe, and severe depression was 20.6%, 23.3%, 22.9%, and 7.2%, respectively. Of the 131 participants (50%) with OAB, 16.8%, 27.9%, and 5.3% reported mild, moderate, and severe symptoms, respectively (Table 1).

Nocturia (*n* = 188, 71.8%), urinary urgency (*n* = 156, 59.5%), daytime frequency (*n* = 93, 35.5%), and urge incontinence (*n* = 76, 29%) were OAB symptoms. There was no statistical difference in frequency, nocturia, and urgency between the sexes, while urge incontinence was higher among females and statistically significant (Table 2).

Table 3 shows that the prevalence of depression (PHQ-9 score ≥ 10) was 53.4%. In the bivariate analysis, single participants were more likely to have depression compared with married participants (crude odds ratio [COR] = 2.14; 95% confidence interval (CI) = 1.30–3.51; *p* = 0.003). In addition, participants with anxiety were more likely to have depression than those without anxiety (COR = 14.41; 95% CI = 7.7–27; *p* < 0.001). In the multivariable analysis, both single (AOR = 1.94; 95% CI = 1.05–3.59; *p* = 0.034), and anxiety (AOR = 14.60; 95% CI = 7.59–28.08; *p* < 0.001) remained statistically significant.

Table 4 shows that the prevalence of anxiety (GAD-7 scores ≥ 10) was 43.9%. In the bivariate analysis, females were more likely to have anxiety compared with males (COR 2.20; 95% CI = 1.25–3.86; *p* = 0.006), single participants were more likely to have anxiety compared with married participants (COR = 2.01; 95% CI = 1.22–3.30; *p* = 0.006), employed participants were less likely to have anxiety compared with unemployed participants (COR = 0.49; 95% CI = 0.27–0.88; *p* = 0.018), underweight participants were more likely to have anxiety compared with normal weight participants (COR = 3.12; 95% CI = 1.28–7.61; *p* = 0.012), and participants with depression were more likely to have anxiety than those without depression (COR = 14.41; 95% CI = 7.7–27; *p* < 0.001). In the multivariable analysis, only females (AOR = 2.57; 95% CI = 1.19–5.54; *p* < 0.016) and depression (AOR = 16.9; 95% CI = 8.42–34.2; *p* < 0.001) remained statistically significant. In addition, students became statistically significant (AOR = 0.37; 95% CI = 0.14–0.94; *p* = 0.038).

Table 5 shows that the prevalence of OAB was 50%. In the bivariate analysis, the median age was higher among participants with OAB than those without OAB (COR = 1.03; 95% CI = 1.00–1.05; *p* = 0.039), students were less likely to have OAB compared with those who were unemployed (COR = 0.46; 95% CI = 0.23–0.92; *p* = 0.027), participants with depression were more likely to have OAB than those without depression (COR = 2.24; 95% CI = 1.37–3.68; *p* = 0.001), and participants with anxiety were more likely to have OAB than those without anxiety (COR = 2.34; 95% CI = 1.42–3.86; *p* = 0.001). In the multivariable analysis, only anxiety (AOR = 2.07; 95% CI = 1.08–3.98; *p* = 0.028) remained statistically significant.

**Figure 2:**
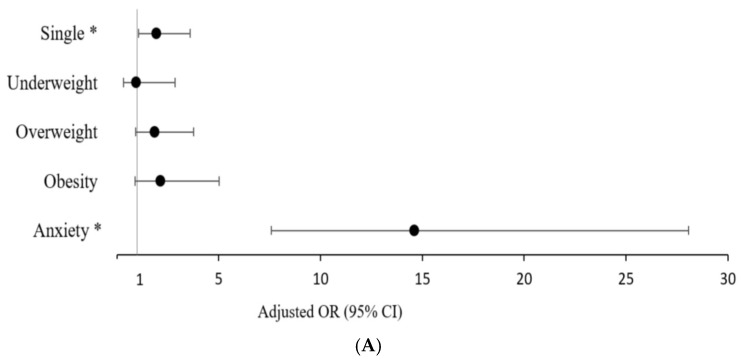

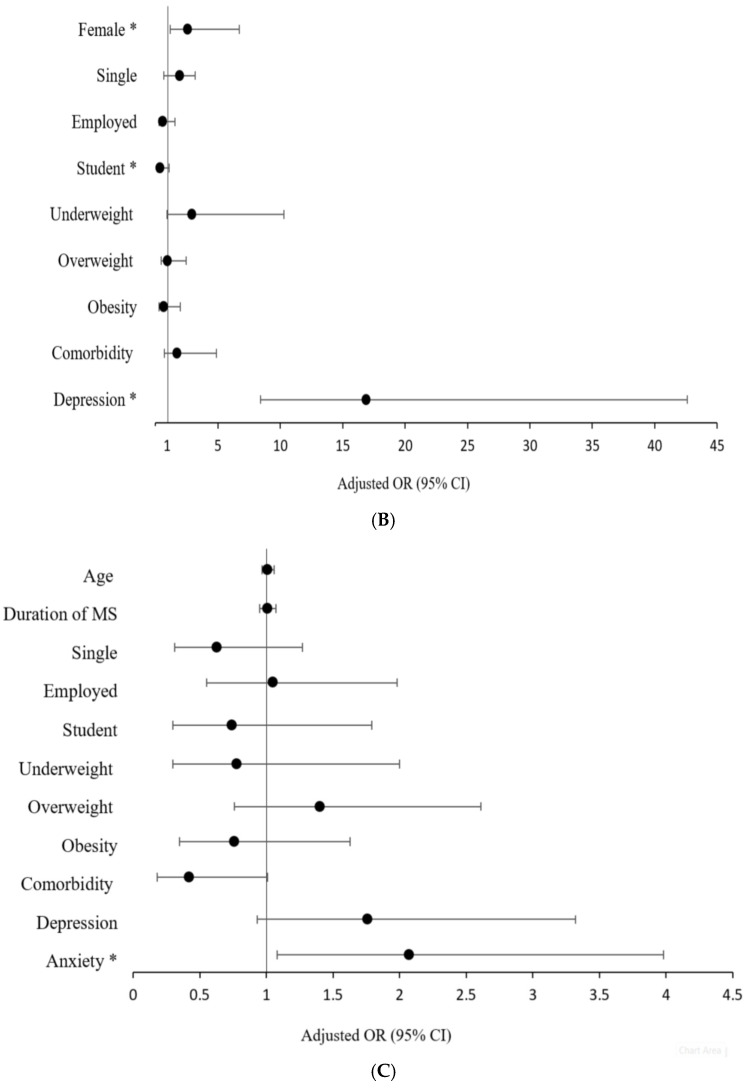
(**A**). Forest plot showing adjusted odds ratios and 95% confidence intervals for factors associated with depression among MS patients. (**B**). Forest plot showing adjusted odds ratios and 95% confidence intervals for factors associated with anxiety among MS patients. (**C**). Forest plot showing adjusted odds ratios and 95% confidence intervals for factors associated with overactive bladder among MS patients. * indicates statistical significance; *p* ≤ 0.05.

Finally, forest plots summarizing the adjusted odds ratios and 95% confidence intervals from the three multivariable analyses (depression, anxiety, and OAB) are presented in Figure 2A–C (* indicates statistical significance; *p* ≤ 0.05).

## 4. Discussion

The main findings of this study showed that OAB, depression, and anxiety were highly prevalent among MS patients in the Qassim region. First, OAB was reported by half of the MS patients, which is consistent with the results of a study conducted in Rome, Italy, and of another multicenter study across four countries (France, Germany, Italy, and the UK) [34,35]. In contrast, research conducted in Iran found less prevalence compared to our study’s results, which may be due to differences in the screening instruments used, as they employed the Actionable Bladder Symptom Screening Tool (ABSST) [36]. A study in Saudi Arabia reported a 94% prevalence of lower urinary tract symptoms (storage, voiding, and postmicturition), but it was executed via an electronic questionnaire administered during the COVID-19 pandemic; thus, many MS patients who were experiencing urinary symptoms were more likely to participate in the study, which might have led to an over-estimation of prevalence [37].

This study found that the most frequent urinary symptom was nocturia, followed by urgency, urinary frequency, and urge incontinence, supporting the findings of a study conducted in the United States, which included more than 9000 MS patients and reported nocturia as the most frequent urinary symptom, followed by urinary urgency and frequency [38]. Our study’s findings also confirm an Iranian study’s report that the predominant urinary tract symptoms in MS patients were nocturia, urgency, diurnal polyuria, and the feeling of incomplete bladder emptying [39]. Another study conducted in Morocco identified various commonly observed symptoms, but urgency and frequency were lower than the rates noted in our study [40]. However, a study conducted in five centers in Turkey reported urinary urgency as the most common symptom, followed by frequency, urge incontinence, and nocturia [41]. These variations in the predominant symptoms may be related to age, duration, severity, and subtype of MS, and differences in sample sizes and measurement tools. The existing literature demonstrates variations in the prevalence of urinary symptoms among MS patients, representing both sexes. A study conducted in Turkey and another in Iran revealed that storage symptoms did not exhibit a significant difference between the two sexes [42,43]. Another study demonstrated urgency as more common in men than in women but found no sex difference in any other urinary symptoms [41]. In our study, there were no sex-related differences in the rates of urgency, frequency, and nocturia; however, urge incontinence was identified as more prevalent among females. Moreover, the distribution of symptom severity among the participants with urinary tract symptoms (mild, moderate, and severe) aligned with the results of a study in Riyadh, noting a similar distribution of the prevalence of urinary tract symptoms among the respondents [37].

In our study, anxiety was significantly higher among MS patients with OAB compared to those without OAB, supporting the findings of a study conducted in Turkey [44]. Additionally, OAB severity and anxiety levels are found to be correlated in MS patients, indicating that higher anxiety is associated with more severe OAB symptoms, as these patients are less likely to adhere to therapies for MS or its complications [45].

Second, the present study found that more than half of the MS patients were identified as having depression. The association between depression and MS is firmly established, with varying levels of prevalence, as reported in different studies [12,19,46,47,48]. In Saudi Arabia, a study conducted in the regions of Riyadh and Jeddah, using the same PHQ-9 scale, reported a prevalence of 42.7%, which is lower than that observed in our study [32]. Likewise, a UAE-based study using the same tool found that 17% of participants met the criteria for depression, which also remains below our reported prevalence [31]. However, the prevalence appears to vary depending on the assessment tools used; for example, another Saudi study based on the Hospital Anxiety and Depression Scale (HADS) criteria reported a prevalence of 58.8% in Riyadh [20].

This study revealed that being single and having anxiety were associated with depression among MS patients. The association between being single and increased depression among MS patients is supported by recent research [49]. In the Saudi context, an existing study has similarly found that widowed or divorced MS patients experienced higher rates of depression, indicating a link between single status and higher depressive symptoms [19]. This observation highlights the protective role of social support in managing the psychological distress associated with MS [16]. The research underscores that social support is vital for emotional well-being, particularly in chronic conditions like MS, where psychological distress is prevalent [32]. Additionally, participants with anxiety were more likely to have depression compared to participants without anxiety. This observation is consistent with findings from a previous study [49].

Third, anxiety affected over two-fifths of the MS patients in our study. Similar to OAB and depression, the prevalence of anxiety shows variability, ranging from 14.9% to 51.1% [12,19,40]. A study conducted in the Riyadh region of Saudi Arabia reported a similar prevalence [19], while a multicenter study conducted in Saudi Arabia reported a lower prevalence when using the GAD-7 scale [32]. Furthermore, a study conducted in a neighboring Arab country reported a lower prevalence compared to our study [31]. Additionally, two studies using different assessment tools in Saudi Arabia reported similar prevalence [20]. In contrast, another study reported a lower prevalence [18].

In our study, female sex, being a student, and having depression were associated with anxiety. Our findings showed that the female sex was linked to a higher level of anxiety, which aligns with studies conducted in Saudi Arabia [19,32,50]. This relationship is further reported on a global level [51,52]. Understanding the gender-specific prevalence of anxiety can help guide targeted therapeutic approaches, emphasizing the importance of addressing mental health in female MS patients [53]. In contrast, being a student was associated with a lower risk of anxiety among MS patients. Research indicated that employment status significantly influenced the psychological well-being of individuals with MS, with unemployment linked to greater perceived stress [52]. Another possible explanation is that students, often engaged in structured environments, receive a higher level of social support [54,55]. The final associated factor was depression, which was associated with a higher level of anxiety compared with those without depression, which aligns with previous studies [17,51,53].

Collectively, the evidence indicates that the reported prevalence of OAB, depression, and anxiety among MS patients varies by study methods, assessment tools, and population characteristics across studies.

Several pathophysiological mechanisms have been proposed to explain the linking of anxiety, depression, and bladder dysfunction in MS patients, including overlapping neuroinflammation, neurodegeneration, autonomic dysfunction, and psychosocial factors [56,57]. However, current evidence mainly demonstrates associations rather than causal relationships. In MS, brain and spinal cord involvement may contribute to cognitive, emotional, and bladder dysfunction, which are interrelated and can worsen quality of life [56,58].

Although this is the first study conducted in the Qassim region, which helps fill the gap in the literature regarding regional variations in Saudi Arabia, our research has some limitations that should be considered. A cross-sectional study design was used, which inherently limits causal inference. This study was conducted at a single tertiary care center, which may limit the generalizability to other populations, settings, and regions. However, it is the only tertiary center in the Qassim region and receives MS patients referred from multiple cities across the region, which may enhance its local relevance and provide a relatively representative sample of MS cases in the Qassim region. Additionally, this study did not include several clinically relevant variables, such as MS subtype, disease severity (e.g., EDSS), relapse activity, treatment status, and disability level, which may influence both psychological and urinary outcomes. Also, anxiety, depression, and OAB were subjectively measured based on self-reported symptoms. We highly recommend carrying out a longitudinal study to understand the complex interplay between OAB, anxiety, and depression among MS patients, with the use of an objective diagnostic tool, as well as considering other relevant symptoms, such as fatigue or motor impairments (e.g., gait disturbances). Finally, incorporating qualitative research can enrich the data by capturing patients’ perspectives and lived experiences of bladder dysfunction, anxiety, and depression, as well as welcoming their willingness to discuss their symptoms with healthcare providers. This wealth of information could provide valuable insights into the psychosocial dimensions of living with OAB, anxiety, and depression among MS patients.

## 5. Conclusions

We conclude that our study identified a high prevalence of anxiety, depression, and OAB symptoms among MS patients, with critical implications for healthcare policy and resource allocation. In response, healthcare institutions and practitioners in the Qassim region have now prioritized screening, early diagnosis and intervention, and integrated care to address these conditions and improve MS patients’ QoL.

## Figures and Tables

**Table 1:** Descriptive characteristics of 262 multiple sclerosis (MS) patients.

Variable	Median	Interquartile Range (IQR)
Age, years	34	26–40
Duration of MS	5	2–9
Overactive Bladder Symptom Score (OABSS)	4	1–7
	**Number**	**Percentage**
Sex		
Female	184	70.2
Male	78	29.8
Marital status		
Married	137	52.3
Single	125	47.7
Occupation		
No	146	55.7
Employed	69	26.3
Student	47	18
Body mass index (BMI)		
Underweight	27	10.3
Normal weight	105	40.1
Overweight	84	32
Obese	46	17.6
Comorbidity		
No	231	88.2
Yes	31	11.8
Anxiety		
No	65	24.8
Mild	82	31.3
Moderate	56	21.4
Severe	59	22.5
Depression		
No	68	26
Mild	54	20.6
Moderate	61	23.3
Moderate to severe	60	22.9
Severe	19	7.2
Overactive bladder (OAB) symptoms		
No	131	50
Mild	44	16.8
Moderate	73	27.9
Severe	14	5.3

**Table 2:** OAB symptoms.

Symptom	Total *n* (%)	Female *n* (%)	Male *n* (%)	*p*-Value
Frequency	93 (35.5)	63 (34.2)	30 (38.5)	0.514
Nocturia	188 (71.8)	132 (71.7)	56 (71.8)	0.993
Urgency	156 (59.5)	113 (61.4)	43 (55.1)	0.343
Urge incontinence	76 (29)	60 (32.6)	16 (20.5)	0.049

**Table 3:** Bivariate and multivariable analyses of factors associated with depression among 262 MS patients.

Variable	Depression		Bivariate Analysis		Multivariable Analysis	
	Yes (*n* = 140)	No (*n* = 122)	COR (95% CI)	*p*-value	AOR (95% CI)	*p*-value
	**Median [IQR]**					
Age, years	33 [26–39]	35 [27–41]	0.99 (0.96–1.01)	0.270		
Duration of MS	5 [2–9]	4.5 [2–10]	0.99 (0.95–1.05)	0.943		
	**Frequency (%)**					
Sex						
Male	41 (52.6)	37 (47.4)	Reference			
Female	99 (53.8)	85 (46.2)	1.05 (0.62–1.79)	0.854		
Marital status						
Single	79 (63.3)	46 (36.8)	2.14 (1.30–3.51)	0.003	1.94 (1.05–3.59)	0.034
Married	61 (44.5)	76 (55.5)	Reference			
Occupation						
No	81 (55.5)	65 (44.5)	Reference			
Employed	37 (53.6)	32 (46.4)	0.93 (0.52–1.65)	0.798		
Student	22 (46.8)	25 (53.2)	0.71 (0.37–1.37)	0.301		
BMI						
Underweight	17 (63)	10 (37)	2.02 (0.85–4.82)	0.114	0.96 (0.32–2.86)	0.939
Normal weight	48 (45.7)	57 (54.3)	Reference			
Overweight	49 (58.3)	35 (41.7)	1.66 (0.93–2.97)	0.085	1.85 (0.91–3.77)	0.092
Obese	26 (56.5)	20 (43.5)	1.54 (0.77–3.10)	0.223	2.13 (0.89–5.03)	0.086
Comorbidity						
Yes	17 (54.8)	14 (45.2)	1.07 (0.50–2.26)	0.868		
No	123 (53.3)	108 (46.8)	Reference			
Anxiety						
No	42 (28.6)	105 (71.4)	Reference			
Yes	98 (85.2)	17 (14.8)	14.41 (7.7–27)	<0.001	14.60 (7.59–28.08)	<0.001

**Table 4:** Bivariate and multivariable analyses of factors associated with anxiety among 262 MS patients.

Variable	Anxiety		Bivariate Analysis		Multivariable Analysis	
	Yes (*n* = 115)	No (*n* = 147)	COR (95% CI)	*p*-value	AOR (95% CI)	*p*-value
	**Median [IQR]**					
Age, years	35 [26–40]	34 [26–39]	1.00 (0.98–1.03)	0.870		
Duration of MS	4 [2–9]	6 [2–10]	0.98 (0.93–1.03)	0.385		
	**Frequency (%)**					
Sex						
Males	24 (30.8)	54 (69.2)	Reference			
Females	91 (49.5)	93 (50.5)	2.20 (1.25–3.86)	0.006	2.57 (1.19–5.54)	0.016
Marital status						
Single	66 (52.8)	59 (47.2)	2.01 (1.22–3.30)	0.006	1.29 (0.66–2.52)	0.454
Married	49 (35.8)	88 (64.2)	Reference			
Occupation						
No	74 (50.7)	72 (49.3)	Reference			
Employed	23 (33.3)	46 (66.7)	0.49 (0.27–0.88)	0.018	0.58 (0.26–1.30)	0.187
Student	18 (38.3)	29 (61.7)	0.60 (0.31–1.18)	0.141	0.37 (0.14–0.94)	0.038
BMI						
Underweight	18 (66.7)	9 (33.3)	3.12 (1.28–7.61)	0.012	2.92 (0.91–9.39)	0.073
Normal weight	41 (39.1)	64 (60.9)	Reference			
Overweight	38 (45.2)	46 (54.8)	1.29 (0.72–2.31)	0.392	0.96 (0.46–1.99)	0.904
Obese	18 (39.1)	28 (60.9)	1.00 (0.49–2.04)	0.992	0.68 (0.28–1.69)	0.410
Comorbidity						
Yes	17 (54.8)	14 (45.2)	1.65 (0.78–3.50)	0.194	1.71 (0.69–4.20)	0.244
No	98 (42.4)	133 (57.6)	Reference			
Depression						
No	17 (13.9)	105 (86.1)	Reference			
Yes	98 (70)	42 (30)	14.41 (7.7–27)	<0.001	16.9 (8.42–34.2)	<0.001

**Table 5:** Bivariate and multivariable analyses of factors associated with OAB among 262 MS patients.

Variable	OAB		Bivariate Analysis		Multivariable Analysis	
	Yes (*n* = 131)	No (*n* = 131)	COR (95% CI)	*p*-value	AOR (95% CI)	*p*-value
	**Median [IQR]**					
Age, years	36 [27–42]	30 [24–38]	1.03 (1.00–1.05)	0.039	1.01 (0.97–1.06)	0.562
Duration of MS	4 [2–10]	5 [2–9]	1.03 (0.98–1.08)	0.249	1.01 (0.95–1.07)	0.812
	**Frequency (%)**					
Sex						
Male	40 (51.3)	38 (48.7)	1.08 (0.63–1.83)	0.787		
Female	91 (49.5)	93 (50.5)	Reference			
Marital status						
Single	55 (44)	70 (56)	0.63 (0.39–1.03)	0.064	0.63 (0.31–1.27)	0.198
Married	76 (55.5)	61 (44.5)	Reference			
Occupation						
No	77 (52.7)	69 (47.3)	Reference			
Employed	38 (55.1)	31 (44.9)	1.09 (0.62–1.95)	0.749	1.05 (0.55–1.98)	0.890
Student	16 (34)	31 (66)	0.46 (0.23–0.92)	0.027	0.74 (0.30–1.79)	0.499
BMI						
Underweight	11 (40.7)	16 (59.3)	0.79 (0.33–1.85)	0.582	0.78 (0.30–2.00)	0.600
Normal weight	49 (46.7)	56 (53.3)	Reference			
Overweight	50 (59.5)	34 (40.5)	1.68 (0.94–3.00)	0.080	1.40 (0.76–2.61)	0.283
Obese	21 (45.7)	25 (54.3)	0.96 (0.48–1.92)	0.908	0.76 (0.35–1.63)	0.474
Comorbidity						
Yes	11 (35.5)	20 (64.5)	0.51 (0.23–1.11)	0.089	0.42 (0.18–1.01)	0.052
No	120 (51.9)	111 (48.1)	Reference			
Depression						
No	48 (39.3)	74 (60.7)	Reference			
Yes	83 (59.3)	57 (40.7)	2.24 (1.37–3.68)	0.001	1.76 (0.93–3.32)	0.083
Anxiety						
No	60 (40.8)	87 (59.2)	Reference			
Yes	71 (61.7)	44 (38.3)	2.34 (1.42–3.86)	0.001	2.07 (1.08–3.98)	0.028

## Data Availability

Data will be available from the corresponding author upon reasonable request.
